# Lacrimal passage irrigation in children with Stevens-Johnson syndrome or toxic epidermal necrolysis: a five-year retrospective study

**DOI:** 10.1186/s12886-018-1014-9

**Published:** 2019-01-18

**Authors:** Qin Xiang, Xu Gao, Jing Fang, Lianhong Pi, Xinke Chen, Lin Chen, Qing Liu

**Affiliations:** 1 Department of Ophthalmology, Children’s Hospital of Chongqing Medical University, Ministry of Education Key Laboratory of Child Development and Disorders, Chongqing, 400014 China; 2 China International Science and Technology Cooperation basis of Child Development and Critical Disorders, Chongqing, China; 3 Chongqing Key Laboratory of Pediatrics, Chongqing, China; 4 Chongqing Engineering Research Center of Stem Cell Therapy, Chongqing, China

**Keywords:** Children, Dexamethasone, Irrigation, Lacrimal systems, Stevens-Johnson syndrome, Toxic epidermal necrolysis

## Abstract

**Background:**

To identity the effect of lacrimal system irrigation in the acute stage in children with Stevens-Johnson syndrome (SJS) or toxic epidermal necrolysis (TEN).

**Methods:**

A retrospective review of 39 patients with SJS or TEN from 2012 to 2017 was analyzed. Patients were divided into two subgroups according to whether they received irrigation in the acute stage. The irrigation group included 21 inpatients and the control group included 18 inpatients. The χ2 test was used to compare clinical findings and data in both groups.

**Results:**

The rate of lacrimal system obstructions in the chronic stage of SJS or TEN in the irrigation group was significantly lower than in the control group (*p* < 0.01). A significant difference was also found between the epiphora rates in patients with SJS or TEN in the chronic stage in the two groups (*p* = 0.047). One of 15 patients with mild dry eyes or without dry eyes in the irrigation group had epiphora, and it affected five of 12 in the control group. The difference between the two groups was significant (*p* = 0.03). Epiphora in the two patients in the control group was long-term, owing to the disappearance of puncta marks.

**Conclusions:**

Lacrimal system irrigation with dexamethasone drops in the acute stage was a simple way to lessen lacrimal system obstructions and epiphora in patients with SJS or TEN. It should be considered a conventional ocular treatment for SJS or TEN.

## Background

Stevens-Johnson syndrome (SJS) and its more severe variant, toxic epidermal necrolysis (TEN), are acute blistering diseases of the skin and mucus membranes. They were first reported in 1922 [1] and 1956 [2], respectively. To date, there has been no unified standard to define the period of the acute stage of SJS or TEN. According to tradition, the first two to 6 weeks after the onset of symptoms is considered the acute stage [3]. Incidences of SJS and TEN are relatively rare, with about 1.2–6 and 0.4–1.2 cases per million persons [4, 5], respectively. However, the two diseases have mortality rates of 1–5% and 25–35% [6, 7], respectively. The underlying etiology of SJS and TEN concentrates on drug use and viral infections, mainly herpes, where 70% of SJS cases and nearly all TEN cases are caused by drug use [8]. To date, more than 100 drugs, including antibiotics, non-steroidal anti-inflammatory drugs (NSAIDs), and anticonvulsants, have been identified as having an association with the development of SJS and TEN [9]. SJS and TEN, involving the skin, mucus membranes, and multiple organs, are potentially life threatening during the acute stage, so more attention is focused on saving the lives of patients, while timely ocular treatment for patients with SJS or TEN is often ignored. Fortunately, with the severe impact of most ocular complications of SJS or TEN, including dry eye and cicatricial ocular blinding, on the quality of patients’ lives in the recovery period of the disease, more light must be shed on the timely ocular treatment of patients with SJS or TEN.

For patients with SJS or TEN, prolonged ulcerations and relentless inflammation in the acute phase might give rise to serious ocular complications. Dry eye; symblepharons; entropion; ectropion; trichiasis; blinding cicatricial ocular surface changes, such as corneal epithelial defects and ulcerations; and corneal or conjunctival scarring are believed the most common ocular complications in patients with SJS or TEN after the initial attack [10]. Traditionally, treatments include the frequent use of artificial preservative-free tears, the removal of pseudomembranes, the lysis of symblepharons using a glass rod, and the use of topical antibiotics and corticosteroids to prevent ocular complications of SJS or TEN. In recent years, amniotic membrane transplantation (AMT) at the bedside has been applied to improve inflammation and promote ocular surface healing during the acute stage of SJS or TEN [11–13]. With a further understanding of the disease and timely ocular treatments, dry eyes would be alleviated in patients with SJS or TEN, lessening the influence of tear secretions.

In addition to the complications mentioned above, lacrimal system obstruction secondary to SJS or TEN is seldom noticed by ophthalmologists, although SJS and TEN have long been recognized as causes of punctal and canalicular obstructions [14]. Dry eyes were so common in the recovery period of SJS or TEN that obstructions of the lacrimal system were often ignored. Many patients with SJS or TEN had dry eyes and few tears, and epiphora secondary to lacrimal system obstruction was often covered by this condition. In extreme cases, the puncta would be obstructed intentionally using punctum plugs in the treatment of severe dry eyes. However, not all patients with SJS or TEN develop severe dry eyes in the recovery period, at least in childhood cases of SJS or TEN in our hospital. In those patients whose excretory ductules of the lacrimal gland were not obstructed by scarring in the fornix and whose tears could be secreted normally from the lacrimal gland, a lacrimal system obstruction would manifest as epiphora. The lacrimal system obstruction would reduce the quality of life and cause discomfort because of the long-term existence of epiphora. This leads us to identify the problem concerning lacrimal system obstructions in patients with SJS or TEN.

Irrigation of the lacrimal system is a conventional way to estimate whether the lacrimal system is obstructed in an ophthalmic clinic. Using dexamethasone to treat a lacrimal passage obstruction could increase the cure rate.

Because some children with SJS or TEN exhibited epiphora in the chronic stage in our clinical practice, we aimed to identify the effect of irrigation of the lacrimal system in the acute stage of SJS or TEN in Chinese children.

## Methods

We retrospectively reviewed the medical case records of 39 patients diagnosed with SJS or TEN and aged below 18 years. All individuals, including 20 males and 19 females, were medical inpatients of the Children’s Hospital of Chongqing Medical University during 2012 to 2017. All patients received systemic treatment after admission. Ocular treatments, including artificial tears, the removal of pseudomembranes, lysis of symblepharons, topical antibiotics, and corticosteroid drops, were also used (see Table 1).

**Table 1:** Clinical findings for patients with SJS or TEN in the irrigation and control groups

	Irrigation group (*n* = 21 cases) (%)	Control group (*n* = 18 cases) (%)	*p* value
Average age	8.14 ± 5.782 years	8.89 ± 6.133 years	NS*
Gender (male: female)	10:11	10:8	NS
Ocular manifestations of patients in the acute stage	pseudomembranes 21(100%)	pseudomembranes 18(100%)	NS
	symblepharon 5(23.81%)	symblepharon 4(22.22%)	NS
Number of patients caused by drug-taking	20 (95.24%)	17 (94.44%)	NS
Associated drugs	antibacterial 8(38.10%)	antibacterial 7(36.84%)	NS
	anticonvulsants 6(28.57%)	anticonvulsants 4(21.05%)	NS
	NSAIDs^a^ 4(19.05%)	NSAIDs^a^ 4(21.05%)	NS
	other 3(14.29%)	other 4(21.05%)	NS
Systemic treatment types	systemic antibiotics and corticosteroids	systemic antibiotics and corticosteroids	
Ocular treatment types in the acute stage	artificial tears, removal of pseudomembranes, lysis of symblepharon, topical antibiotics and corticosteroids drops, irrigations of lacrimal system with dexamethasone drops	artificial tears, removal of pseudomembranes, lysis of symblepharon, topical antibiotics and corticosteroids drops	

*NS No significance

^a^*NSAIDs* Non-Steroidal Anti-inflammatory Drugs

The inclusion criteria were (1) patients with a diagnosis of SJS or TEN in the acute stage, (2) patients whose eyes had pseudomembranes and who received an ophthalmologic consultation, (3) patients who underwent fluorescein sodium staining of the cornea, (4) patients in whom Schirmer’s test and break-up time (BUT) were performed in the fourth week of the acute stage, and (5) patients who underwent irrigation during the chronic stage of SJS or TEN to aid in the identification of whether the lacrimal system was obstructed.

The acute stage was based on the acute onset of a high fever, a serious mucocutaneous illness with skin eruptions, the involvement of at least two mucosal sites, and the pathologic findings of a skin biopsy that demonstrated necrotic changes to the dermis. The definition and classification of dry eye was according to Michael et al. [15]. Because irrigation of the lacrimal passage was not the conventional treatment for SJS and TEN, in our clinical work, we usually irrigated patients once pseudomembranes covered their puncta.

Patients were divided into two subgroups according to whether they received irrigation in the acute stage. Subjects (*n* = 21) having received lacrimal passage irrigation with dexamethasone drops twice a week for 4 weeks while in the acute stage were defined as the irrigation group, while subjects (*n* = 18) who received no irrigation in the acute stage were defined as the control group.

Irrigation of the lacrimal passage was performed in office-based and operating room settings. Topical anesthetic drops with 0.4% oxybuprocaine hydrochloride (Santen Pharmaceutical Co., Ltd.) were applied to the eye(s) three times in intervals of 2 min. Then, a 5-ml syringe with 0.3% tobramycin and 0.1% dexamethasone ophthalmic drops (1–1.5 mL, Tobradex ®, Alcon, Fort Worth, USA) was irrigated through the upper canaliculus to avoid injury to the lower canaliculus. If the lower punctum was covered by pseudomembranes, irrigation was performed through both the upper and lower puncta. The nasolacrimal duct was unobstructed when the solution created no reflux and the patient swallowed the solution through the throat. The nasolacrimal duct was defined as obstructed when the irrigation solution was refluxed through the upper or lower punctum. In addition, epiphora was determined if the discharge of tears was blocked and confined in the eyes. We identified no complications from this procedure.

The SPSS (version 17.0) software was used to perform all statistical calculations. The mean ages at the first treatment were expressed as the mean ± standard deviation (S.D.) and they were compared using the *t* test. The χ2 test was used to compare clinical findings and data with or without irrigation of a lacrimal system using dexamethasone drops in both groups, where *p* < 0.05 was considered statistically significant.

## Results

### Clinical findings for patients with SJS or TEN in the irrigation and control groups

As shown in Table 1, 39 patients were studied, including 20 males and 19 females whose ages averaged 8.14 ± 5.782 years in the irrigation group and 8.89 ± 6.133 years in the control group. In total, 37 patients (20 in the irrigation group and 17 in the control group) had a clear history of drug use, including antibacterial drugs, anticonvulsants, and NSAIDs, and the other two cases were caused by a viral infection. All patients with SJS or TEN in both groups were in the first week of the acute stage when they arrived at the hospital. Nine patients (five in the irrigation group and four in the control group) had corneal damage, while 12 patients (seven in the irrigation group and five in the control group) had conjunctiva involved. Lid margin sloughing happened in three cases in both groups. There was no significant difference in the percentages of clinical findings between both groups.

### A comparison of the data of patients with or without irrigation of the lacrimal system using dexamethasone drops between two groups

Table 2 shows the data of patients with or without irrigation of the lacrimal system using dexamethasone drops in the chronic stage in both groups. Only two of 21 (9.52%) patients in the irrigation group were found to have lacrimal system obstructions, compared to 14 of 18 (77.78%) patients in the control group. The rate of obstructions to the lacrimal system in the chronic stage of SJS or TEN in the irrigation group was lower than in the control group, and the rates in both groups differed significantly (*p* < 0.01). One of 21 (4.76%) patients in the irrigation group had symptoms of epiphora compared with five of 18 (27.78%) cases in the control group (*p* = 0.047). None of the cases showed complete disappearance of the puncta marks from the eyelids of the inner canthus in the irrigation group compared with two cases in the control group, but there was no statistical difference.

**Table 2:** A comparison of clinical findings in the chronical stage between two groups

clinical findings		Irrigation group (n = 21) (%)	Control group (*n* = 18) (%)	*P* value
obstructions of lacrimal systems		2 (9.52%)	14 (77.78%)	*p* < 0.01
epiphora		1 (4.76%)	5(27.78%)	0.047
marks of puncta disppeared		0	2 (11.11%)	NS
severe dry eyes		6(28.57%)	6(33.33%)	NS
mild dry eyes or without dry eyes		15(71.43%)	12(66.67%)	NS
epiphora	in severe dry eyes	0	0	
	in mild dry eyes or without dry eyes	1(6.67%)	5(41.67%)	0.03

In the chronic stage, the number of patients with severe dry eyes was six in both groups. The number with mild dry eyes or without dry eyes was 15 (71.43%) in the irrigation group and 12 (66.67%) in the control group. The rates of severe dry eyes were 28.57% in the irrigation group compared with 33.33% in the control group. There was no significant difference concerning the previous rates between the two groups. One of 15 (6.67%) patients with mild dry eyes or without dry eyes in the irrigation group had epiphora, while five of 12 (41.67%) had epiphora in the control group, showing significant statistical differences (*p* = 0.03).

## Discussion

In this study, we observed the effect of irrigation of the lacrimal system with dexamethasone drops for SJS and TEN during the last 5 years. The rate of lacrimal system obstruction in the chronic stages of SJS or TEN in the irrigation group was much lower than in the control group (*p* < 0.01). A significant difference was also found between two groups in the rates of epiphora during the chronic stage (*p* = 0.047).

SJS and TEN are acute immunological mucocutaneous disorders involving the mucosa of the whole body, as part of the mucosa—the mucosa of the lacrimal system—is also involved in the acute stages of SJS or TEN. As dry eyes and blinding cicatricial ocular surface changes are highly common complications of SJS or TEN, ophthalmologists have always focused on them, paying little attention to lacrimal system obstructions secondary to SJS or TEN [10]. In our clinical observation, we noticed that lacrimal system obstructions were not a rare complication in children. In total, 14 of 18 (77.78%) patients in the control group were found to have lacrimal system obstructions in the chronic stage, and five had a long period of epiphora from one to 2 months after the acute stage of SJS or TEN. Lacrimal system obstructions in the chronic stage were also found in the irrigation group in two of 21 (9.52%) cases. The results suggested an association between irrigation of the lacrimal system with dexamethasone drops and a decreased rate of lacrimal system obstructions.

In this study, we also found a significant difference between the rates of epiphora in patients with SJS or TEN in the chronic stage in both groups. Furthermore, the difference in rates concerning epiphora in patients with mild dry eyes or without dry eyes in the two groups was found to be significant. It was normal that patients with severe dry eyes in the chronic stage of SJS and TEN had no epiphora. Because the impact on the secretion of tears in patients with mild eyes or without dry eyes was lesser than in patients with severe dry eyes, lacrimal system obstructions might lead to epiphora in patients with mild dry eyes or without dry eyes. With the development of therapy methods to treat SJS and TEN, including AMT at the bedside, and more prompt treatment of the eyes, fewer impacts on the secretion of tears and the ocular surface will occur in patients with SJS and TEN in the chronic stage [11, 16]. Most patients with SJS or TEN may avoid developing severe dry eyes in the chronic stage in the future. As a result, lacrimal system obstructions may cause more patients with mild dry eyes or without dry eyes to develop epiphora. Interestingly, the lower epiphora rate in patients with mild dry eyes or without dry eyes in the irrigation group was in line with the lower rate of lacrimal system obstructions in the chronic stage in the irrigation group. This might be because lacrimal system obstructions were associated with epiphora. Further studies are needed to support this hypothesis.

Irrigation of the lacrimal system with tobramycin and dexamethasone drops in the acute stage has at least three advantages. On the one hand, irrigation of the lacrimal system with dexamethasone drops may reduce adhesions in the mucosa of the lacrimal system involved in SJS or TEN. The treatment seems to decrease the rate of lacrimal system obstructions in the chronic stage in our clinical observation. For patients with mild dry eyes or without dry eyes in the chronic stage, a decreased rate of lacrimal system obstructions means a lessened chance of developing epiphora. On the other hand, irrigation can make the nasolacrimal duct unobstructed by the flush pressure, and timely irrigation can prevent occlusion of the puncta caused by pseudomembranes. If the occlusion is not discovered soon enough, the organization of pseudomembranes will emerge over time. Puncta marks in the palpebral margin would probably be found with difficulty. Besides, using tobramycin during the operation could reduce intraoperative infections. In two cases in the control group, the puncta marks disappeared completely from the eyelids of the inner canthus during the second month after the acute stage of SJS or TEN. The benefit of syringe use is the combination of physical irrigation, steroids, and antibiotics. Mechanical lavage may be involved in the physical flush pressure of irrigation and the anti-inflammatory and anti-infective effects of eye drops. Therefore, it was better to initiate the first irrigation as soon as the patients were in a stable condition.

Irrigation with dexamethasone worsens the risk of dry eye/corneal disease. Usually, moderate–severe SJS/TEN cases need to accept systemic glucocorticosteroid treatment regardless of whether the patients had eye damage. In this study, however, not all patients recorded worsened dry eye/corneal disease through irrigation with dexamethasone. This result may be due to a small number of subjects. In our further study, we need to observe more patients to confirm this question.

It should be noted that irrigation of the lacrimal system with dexamethasone drops has certain limitations that must be considered when contemplating its contributions. First, the operation is not needed in those with serious dry eyes. In some cases, we would prefer lacrimal system obstructions to avoid severe dry eyes. Second, in the initial stage of acute SJS or TEN, irrigation of the lacrimal system is improper except for in stable patients. Third, a relatively high incidence of epiphora was found in Chinese children with SJS and TEN, though it is unclear whether Chinese adults or patients with SJS and TEN in other areas of the world have the same incidence of epiphora. If epiphora in these patients does not occur, irrigation of the lacrimal system is not necessary. More cases are needed to clarify this point. Fourth, in this retrospective study, all cases involved had eye damage, and moderate–severe SJS/TEN cases were more likely to have eye damage, so we need to collect more samples, including milder patients with eye damage, to avoid bias. Finally, because SJS and TEN in the vast majority of our patients were caused by drug use, viral infection was identified as an etiologic factor associated with few patients. Therefore, topical corticosteroids should be used cautiously to prevent ocular scar formation or adhesions in the mucosa of the lacrimal system in patients whose SJS or TEN was caused by a viral infection.

## Conclusion

Lacrimal system obstructions are one of the most common ocular complications of SJS and TEN in Chinese children patients. With increased attention to ocular complications and the development of treatment in the early stage of SJS and TEN, the incidence of excretory ductule obstruction of the lacrimal gland by scarring in the fornix in the chronic stages of SJS and TEN would be reduced. The incidences of further worsened epiphora would increase owing to the ignorance of lacrimal system obstructions. Irrigation of the lacrimal system with dexamethasone drops can not only reduce the incidence of lacrimal system obstructions in the chronic stages of SJS and TEN, but it can also prevent the disappearance of puncta marks. It seems an effective, safe, easy-to-perform, and minimally invasive technique to lessen lacrimal system obstructions and epiphora in patients with SJS or TEN.

## Abbreviations

AMT: Amniotic Membrane Transplantation
BUT: Break-Up Time
NSAID: Non-Steroidal Anti-inflammatory Drugs
SJS: Stevens-Johnson syndrome
TEN: Toxic Epidermal Necrolysis
